# Pneumatic Balloon Dilatation for Achalasia Cardia; Early & late results, a single center study

**DOI:** 10.12669/pjms.335.13685

**Published:** 2017

**Authors:** S. Waqar H. Shah, Arshad K. Butt, K. Malik, Altaf Alam, Anwaar A. Khan

**Affiliations:** 1 Dr. S. Waqar H. Shah, MBBS. Department of Gastroenterology-Hepatology, Shaikh Zayed Medical Complex, Lahore, Pakistan; 2 Dr. K. Malik, FCPS. Department of Gastroenterology-Hepatology, Shaikh Zayed Medical Complex, Lahore, Pakistan; 3 Dr. Arshad K. Butt, FCPS. Department of Gastroenterology-Hepatology, Shaikh Zayed Medical Complex, Lahore, Pakistan; 4 Dr. Altaf Alam, FRCP. Department of Gastroenterology-Hepatology, Shaikh Zayed Medical Complex, Lahore, Pakistan; 5 Dr. Anwaar A. Khan, M.D, MACP, FACG, FRCP. Department of Gastroenterology-Hepatology, Shaikh Zayed Medical Complex, Lahore, Pakistan

**Keywords:** Idiopathic Esophageal Achalasia, Lower esophageal sphincter, Pneumatic balloon dilatation

## Abstract

**Objective:**

Achalasia Cardia is treated by Pneumatic balloon dilatation, Heller’s Myotomy and recently, by Peroral Esophagaeal Myotomy. This study reports the efficacy of pneumatic balloon dilatation as a non-surgical motility in achieving relief of dysphagia, clinical improvement and recurrence. Long-term complications were reported.

**Methods:**

Eight hundred ninety two adult achalasia patients of both genders were treated from January 1988 till December 2011, with pneumatic balloon (Rigiflex Microvasive^®^) dilatation, under fluoroscopy Barium swallow was obtained prior to and five minutes after dilatation to evaluate for efficacy of dilatation as well as for complications. Patients not responding to 30 mm balloon had repeat dilatation with 35 mm balloon after 8 weeks. All patients were enrolled in regular follow up at one, six months and yearly intervals up to a period of five years. Recurrence was defined as an increase in symptom score at 8 weeks greater than 50% of their baseline value. These patients were treated with 35 mm balloon or referred for surgical intervention.

**Results:**

Of 892 patients, follow up was obtained in 50% for 5 years, 9.2% for 4-years), 9.3% for 3-years, 10% for 2-years and 21.5% for 1-year of patients. One patient died after repeat dilatation. Eighty-eight patients were excluded from this analysis (20 died due to non-procedure related causes and another 68 were lost during follow up). Statistically significant improvement was noted in reduction in height and width of barium column and symptom score coupled with weight gain during follow up. Forty-eight patients were subjected to repeat dilatation with 35 mm balloon, two of these developed post-procedure perforations with one mortality. Three non-responsive patients required surgical laparoscopic myotomy. No carcinoma of esophagus was reported during follow up. One patient post dilatation, developed esophageal bezoar. A single pneumatic dilatation achieved a remission rate of 93% at four years, 90% at three years, 95% at two years and 92% at one year post dilatation.

**Conclusion:**

Achalasia of esophagus can be effectively and safely treated with balloon dilatation to achieve adequate short and long-term symptomatic relief with a low complication rate.

## INTRODUCTION

Idiopathic achalasia of esophagus is a motility disorder of uncertain etiology which presents as dysphagia, night regurgitation, vomiting, weight loss and chest pain.

Pathophysiologically, degeneration of myenteric plexus leads to high amplitude non-peristaltic contractions (vigorous achalasia) due to unopposed action of excitatory neurotransmitters. Progressive loss of cholinergic neurons over time, results in dilatation and low amplitude simultaneous contractions in the esophageal body (classic achalasia).

Treatment options which include pneumatic dilation, surgical myotomy and Per-oral endoscopic myotomy (POEM) disrupt the integrity of lower esophageal sphincter (LES) to achieve symptom resolution, promote esophageal emptying and preventing progression to mega esophagus.^1^,^2^ Pneumatic dilatation can be offered to all age groups, as an outpatient procedure, under conscious sedation and early return to work. There may be mild if any occurrence of gastroesophageal reflux disease (GERD).

Achalasia suspected on history, is confirmed by barium swallow, esophageal endoscopy and esophagealmanometry. The disease usually seen in 3^rd^ to 6^th^ decade is most common in 4^th^ decade. It has been reported from birth till even the 9^th^ decade.^1^ Reported estimates vary from 0.4 to 1.1 per 100,000 for incidence and 7.9 to 12.6 per 100,000 for prevalence.^3^

Circular muscle fibers of LES are disrupted by balloon guided forceful dilatation thereby overcoming functional obstruction of sphincter. Although, a number of balloons are available and used for pneumatic dilatation (Brown McHardy, Hurst Tucker, Mosher and Rider Moller), we prefer Rigiflex pneumatic balloon manufactured by Microvasive, Watertown, MA. These balloons are used either in a non-graded protocol or a gradually increasing size starting with the smallest balloon if results are considered non-satisfactory.^3^,^4^

An observational study of 300 achalasia patients treated with pneumatic balloon dilatation with a median follow up of 3.8 years was reported from our centre in 2005.^5^ Corollary to this, additional 592 patients were enrolled with 1 year follow up intervals, until the fall of 2011, total of 5 years follow up.

### This study aimed to assess

1. Duration of clinical remission following dilatation.
2. To evaluate for possible complications i.e. perforation and post dilatation GERD, particularly with the use of larger 35 mm balloon diameter.
3. Any occurrence of cancer of the esophagus as reported in other studies.^6^,^7^

## METHODS

Achalasia suspected on symptoms of dysphagia, vomiting, regurgitation, loss of weight was confirmed by combination of tests i.e. barium esophagogram, upper GI endoscopy, and esophageal manometry. Patients with secondary achalasia and those deemed unfit to tolerate endoscopic procedures, due to severe co-morbid conditions, were excluded from this study. All patients gave informed consent. Ethical approval for the study was granted by Hospital Administration since no formal Institutional Review Board existed at that time and all studies requiring ethical approval were processed by the Hospital Administrator/Assistant Administrator.

As described in our previous report,^4^ symptom severity for dysphagia, night cough, chest pain and heartburn were recorded on a 4-point scale for each symptom (asymptomatic = 0, 1 = < 1 per month, 2 = several times per month, 3 = several times in a week and 4 = daily). Follow up was arranged at one and six months and then yearly after index dilatation. Patients were instructed to maintain a diary of their symptoms which was reviewed at each visit by the physician. At each visit scores reported by patients for individual symptoms were added to arrive at a composite symptom score. A reduction in composite symptom score of at least 50% compared with pre-treatment score was considered as evidence of clinical remission whereas a score greater than 50% at any time in follow up was deemed as relapse.

Barium esophagogram done five minutes after pneumatic dilatation was evaluated by a consultant radiologist for any tear or perforation, 50% reduction in height of baseline versus five minute post-dilatation esophageal barium column was considered effective emptying. Width of barium column was used to classify esophagi for dilatation as mild, 3.1-4.9 cms, moderate 5-6.9 cms, marked 7 to 8.9 cms and massive, more than 9 cms. Last group was excluded from the present study.

Esophageal manometric studies were performed by two experienced physicians (A.A.K and S.W.H.S) as described earlier.^5^ Manometric criteria required demonstration of absence of peristalsis of the esophageal body, incompletely relaxing lower esophageal sphincter (LES) with a residual pressure > 5 mm Hg, and gastric pressure less than the esophageal pressure.^8^

**Table-I:** Grading system for evaluation of clinical symptoms.

*Symptoms/Score*	*0*	*1*	*2*	*3*
Dysphagia to solids	none	Weekly	daily	each meal
Dysphagia to liquids	none	Weekly	daily	each meal
Active regurgitation	none	Weekly	daily	each meal
Passive regurgitation	none	Weekly	daily	daily
Chest pain	none	Weekly	daily	daily

### Technique of balloon dilatation

Balloon dilatation was initiated, under fluoroscopy, with 30 mm balloon, relapsers were re-dilated with a 35 mm balloon, and non-responders were offered surgical intervention. The method of dilatation has been described earlier.^5^ Balloon distension time to obliterate waist, was six seconds since 1998, and 60 seconds from 1988 till 1998, inasmuch as, equal efficacy of 60 seconds versus six seconds was reported in our earlier study in 1998.^9^

On Post-dilatation, all patients were observed for 12 hours for complications, and subsequently followed up at the GI out-patient clinic at 1and 6 months and then yearly intervals. In this study, Microvasive Rigiflex pneumatic balloon (Watertown, MA) was used for its ease. Rigiflex balloons are 10 cm long, with diameter, of 30, 35, and 40 mm. Over inflation, does not increase the diameter of balloon but results in longitudinal rupture at approximately 20 psi (1,050 mm Hg).

### Statistical analysis

Demographic data was recorded as frequency/percentage while continuous variables were reported as Mean ± SD. Comparison of baseline, one, six months and yearly symptom scores recorded during follow up was done employing Friedman’s 2-Way ANOVA. Both composite score at baseline, one, six months and yearly follow up of esophageal barium height and width at baseline and five minute post dilatation were compared by Wilcoxon matched pairs signed rank test. All data was analyzed with IBM SPSS version 20 with significance level at < 0.05.

## RESULTS

This study reports post dilatation follow up of 892 patients for up to five years. The demographic features and presenting symptoms are shown in Table-II. Symptomatic relief was achieved with dilatation using 30 mm balloon in 844 patients documented by serial improvement in mean composite symptom scores at 1month (2.2 ± 0.46), 6 month (1.6 ± 0.7) and 12 month (1.6 ± 0.3) follow up, compared with baseline (11.3 ± 1.9). Data for change in symptom score at each visit analyzed using Friedman’s 2-Way ANOVA test appears in Table-III. Table-IV presents pair-wise comparison of data for barium height, width and patient weight based on Wilcoxon test. Barium column height decreased significantly from 13.8 ± 6.2 cm pre-dilatation to 7.5 ± 3.4 cm post dilatation (p <0.05). Width of barium column decreased from 4.9 ± 2.3 cm to 3.1 ± 3.6 cm post dilatation (p<0.05). Mean patient weight improved from baseline value of 52.1 ± 6.7 kg to 59.2 ± 5.4 kg and 67.4 ± 5.3 kg at 6 and 12 months respectively, post-dilatation (p<0.05).

**Table-II:** Demographic features and presenting symptoms.

Sex (M/F)	5 12/380 (Ratio 1.34:1)
Mean age at time of diagnosis (range)	46.7 yr± 14.01 (18-67)
Mean duration of symptoms atdiagnosis (range)	27 months (2-98)
Regurgitation/vomiting	87%
Dysphagia	89%
Weight loss>2.0 kg or inability to gain weight	16%
Recurrent pneumonia	7%
Nocturnal cough	29%
Heartburn	7%

**Table-III:** Symptom Scores at baseline, 1, 6 and12 months of follow up.

	*Mean ± SD*			

*Symptoms*	*Baseline*	*1 month*	*6 month*	*12 month*
Dysphagia	4.3±1.3	1.3±0.4	1.4±0.5	1.1±0.7
Chestpain	0.7± 1.2	0.4±0.4	0.0	0.0
Regurgitation	2.7±0.6	0.0	0.0	0.0
Night cough	1.7± 0.3	0.0	0.0	0.0
Heartburn	1.6 ± 0.4	0.5 ± 0.3	0.2 ± 0.6	0.5 ± 0.4
Composite score	11.3 ± 1.9	2.2 ± 0.4	1.6 ± 0.4	1.6 ± 0.3

Following analyses are based on Friedman’s ANOVA test: Composite symptom score basal vs. l month, basal vs. 6 months and basal vs 12 months p <0.05. Composite symptom score 1 month vs 6 and 12 months and 6 months vs 12 months p >0.05.

**Table-IV:** Changes in barium parameters and weight of patients.

*Parameter*	*Mean*	*± S.D*
***Barium studies***		
a. Barium height, pre-dilatation (cm)	13.8	6.2
b. Barium height, 5 minute post-dilatation (cm)	7.5	3.4
c. Barium width, pre-dilatation (cm)	4.9	2.3
d. Barium width, 5 minute post-dilatation (cm)	3.1	1.6
***Weight gain***		
e. Weight, baseline (kg)	52.1	6.7
f. Weight, 6 months (kg)	59.2	5.4
g. Weight, 12 months (kg)	67.4	5.3

Following analyses are based on Wilcoxon test: Barium height pre and post-dilatation p <0.05 Barium width pre and post-dilatation p <0.05 Weight gain basal vs 6 months p <0.05

Forty eight patients (29 males and 19 females) did not achieve adequate symptom relief and were subjected to repeat dilatation Three of these did not achieve adequate symptom relief, hence, were referred for laparoscopic myotomy.

### Immediate and late complications of dilatation

Post dilatation perforation with 35 mm balloon was noted in two of 892 patients. One responded to medical management whereas, the second patient, a 72 years old diabetic lady with chronic bronchitis who underwent surgery, died of sepsis. Esophageal bezoar was noted in one patient during follow up evaluation, of acute onset of dysphagia and was treated endoscopically.^10^

Troublesome reflux symptoms were reported and medically treated in 52 patients (5.8%), 19 patients after first dilatation and 33 after second dilatation with larger, 35 mm balloon. Twenty one patients died from unrelated causes on follow up.

### Follow up data

Follow up data of patients is presented in Table-V. Of 892 patients, follow up was obtained in 50% for 5 years, 9.2% for 4-years, 9.3% for 3-years, 10% for 2-years and 21.5% for 1-year. Symptom score recorded at each follow up visit showed a significant improvement in mean composite scores, compared with baseline. This improvement was sustained during follow up visits.

**Table-V:** Change in Composite symptom score up to 5 years follow up.

*Time of evaluation*						

*Follow up period*	*Baseline*	*1st-year*	*2nd-year*	*3rd-year*	*4th-year*	*5th-year*
5-years n = 416	11 ± 1.9	1.6 ± 1.2	1.4 ± 0.6	1.4 ± 0.5	1.0 ± 0.5	1.0 ± 0.4
4 years n = 69	11.4 ± 1.5	1.3 ± 0.8	1.1 ± 0.6	1.0 ± 0.6	1.3 ± 0.5	-
3 years n = 62	9.0 ± 2.6	2.0 ± 1.0	2.5 ± 0.8	4.0 ± 1.1	-	-
2 years n = 81	10.4 ± 2.2	3.9 ± 1.9	2.3 ± 0.9	-	-	-
1 year n = 176	10.5 ± 2.7	3.2 ± 0.9	-	-	-	-

Total patients enrolled = 892, Lost to follow up = 68, Died = 20, Total patients in follow up = 804.

## DISCUSSION

Definitive cure for achalasia is elusive; but significant symptomatic relief can be achieved. There are several effective therapeutic options including pneumatic dilatation either by one time procedure or graded dilatation. Alternatively, laparoscopic myotomy or POEM are offere.^2^ The choice between endoscopic and surgical procedures is dictated by local expertise and facilities. Early achalasia with dysphagia as principal symptom with normal endoscopy is often confused with gastroesophageal reflux disease. Esophageal manometry helps in early diagnosis. This, however, needs a high index of suspicion since vomiting, weight loss and nocturnal aspiration may be absent or occur intermittently at an early stage; hence the delay in diagnosis.^11^

All presently available treatment options aim to improve esophageal emptying with symptomatic relief. None can restore the irreversible esophageal aperistalasis. Balloon dilatation, surgical myotomy and POEM all disrupt the integrity of circular muscle fibers of LES resulting in decreased resistance to esophageal emptying aided by gravity. Botulinum toxin (Botox^®^) injected at the LES also accomplishes the same result albeit, temporarily.^12^ Calcium channel blockers and nitrates may provide transient relief, particularly, in vigorous achalasia.^13^

Other studies showed efficacy of balloon dilatation varying from 86 to 100%.^3^,^14^,^15^ In their large prospective study, Barkin et al. reported success rate of 90%^16^ using 35 mm balloon. They reported short hospitalization or same day outpatient procedure with significant cost reduction without increase in complications. Although, potential for perforation is low, there were two cases of perforation in our study. Vaezi et al. reported two esophageal perforations in 10 year follow up, with a 3.0 cm balloon.^13^ Echardt et al., reported remission of symptoms in 59% on 1year and 26% on 5 year follow up.^17^ Our results concur with published data, reporting success in 65 to 69% cases after a single dilatation, increasing to 77% after re-dilatation.^17^-^19^

The difference in results among studies reported, may be attributed to the types of balloons used, we used 30 & 35 mm Microvasive Balloon for its safety, as complications have been reported with larger size.^11^

Balloon distension time varies across the studies from 15-60 seconds. In our earlier study, we compared six seconds vs 60 seconds in establishing safety and efficacy, both were found comparable.^9^ Therefore, in this study 6 second distention time was selected after the study report. Some investigators only performed a single dilation^17^ but most employed an incremental protocol with initial dilatation doneusing 30 mm, followed if required by progressively larger 35 and 40 mm balloons.^14^ Some centers from Europe have reported success with serial incremental dilations with the LES pressure less than 10–15 mm Hg as end point.^14^,^16^,^20^ We kept a 4-6 week follow up schedule in esophageal emptying studies, as reported by other centers.^14^,^20^,^21^

Serious complications were infrequent, in this study. Of 892 patients, two patients developed perforation of esophagus after using 35 mm balloon. Vela et al have reported incidence of perforation up to 5%.^22^ Difficulty in maintaining the balloon in position at LES during the procedure is considered potential risk factor for perforation^23^ but we did not encounter this complication on first dilatation. Others have reported higher risk of perforation with 35 mm balloon than a 30 mm balloon.^24^

Minor problems of gastro esophageal reflux were seen in 52 patients (9.7%) in our study, managed with antireflux measures, whereas others reported up to 15-35%.^25^ A meta-analysis of 24 studies of 1144 patients reported in 2009 by the same authors, with a mean follow-up of almost three years, using Rigiflex pneumatic balloon dilator showed, good to excellent symptom relief in 74 to 90% of patients using 30, 35 and 40 mm balloons, respectively.^25^

In some studies, about 30% experienced relapse after 4-6 year follow-up.^26^,^27^ Rarely, esophageal bezoar may present as a minor complication in achalasia and can be managed endoscopically.^10^,^28^

## CONCLUSION

This report indicates that symptomatic and objective relief, with a low complication rate can be achieved with pneumatic balloon dilatation in achalasia. We recommend it as the procedure of first choice in adults, preferably with 30mm ballon at outset for its safety, when equipped with the requisite skills. Whereas, surgical intervention or POEM may be offered as alternative therapy in achalasia or who fail to respond to balloon dilatation.
